# Supplementary material for: Cryo-EM structure of the volume-regulated anion channel LRRC8D isoform identifies features important for substrate permeation
Source: Commun Biol. 2020 May 15;3:240. doi: 10.1038/s42003-020-0951-z (PMC7229184; doi:10.1038/s42003-020-0951-z)
Supplement: Supplementary file 1 — Supplementary Information [file 42003_2020_951_MOESM1_ESM.pdf]

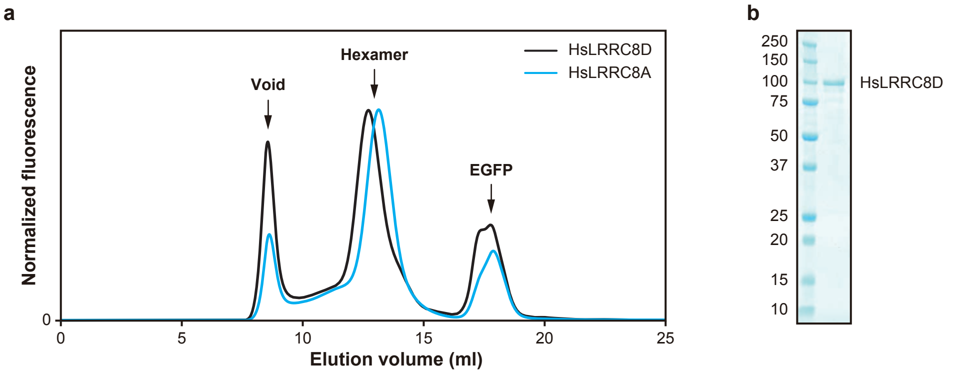

c

Identified proteins	Abundance
volume-regulated anion channel subunit LRRC8D [Homo sapiens]	1.11E+10
hCG1992406, isoform CRA_a [Homo sapiens]	1.74E+09
Chain B, Structure Of Appbp1-Uba3~nedd8-Nedd8-Mgatp-Ubc12(C111a), A Trapped Ubiquitin-Like Protein Activation Complex	9.22E+08
Valosin-containing protein [Homo sapiens]	7.08E+08
heat shock cognate 71 kDa protein isoform 1 [Homo sapiens]	6.97E+08
tubulin beta-4A chain isoform 1 [Homo sapiens]	3.45E+08
unnamed protein product [Homo sapiens]	5.19E+08
Chain A, Crystal Structure Of A Heat Shock 70 kda Protein 2 (hspa2) From Homo Sapiens At 1.80 A Resolution	3.68E+08
78 kDa glucose-regulated protein precursor [Mus musculus]	5.46E+08
heat shock 70kDa protein 1-like [Homo sapiens]	2.08E+08
heat shock protein 60 [Homo sapiens]	3.80E+07
ATP synthase subunit beta, mitochondrial precursor [Homo sapiens]	2.86E+08

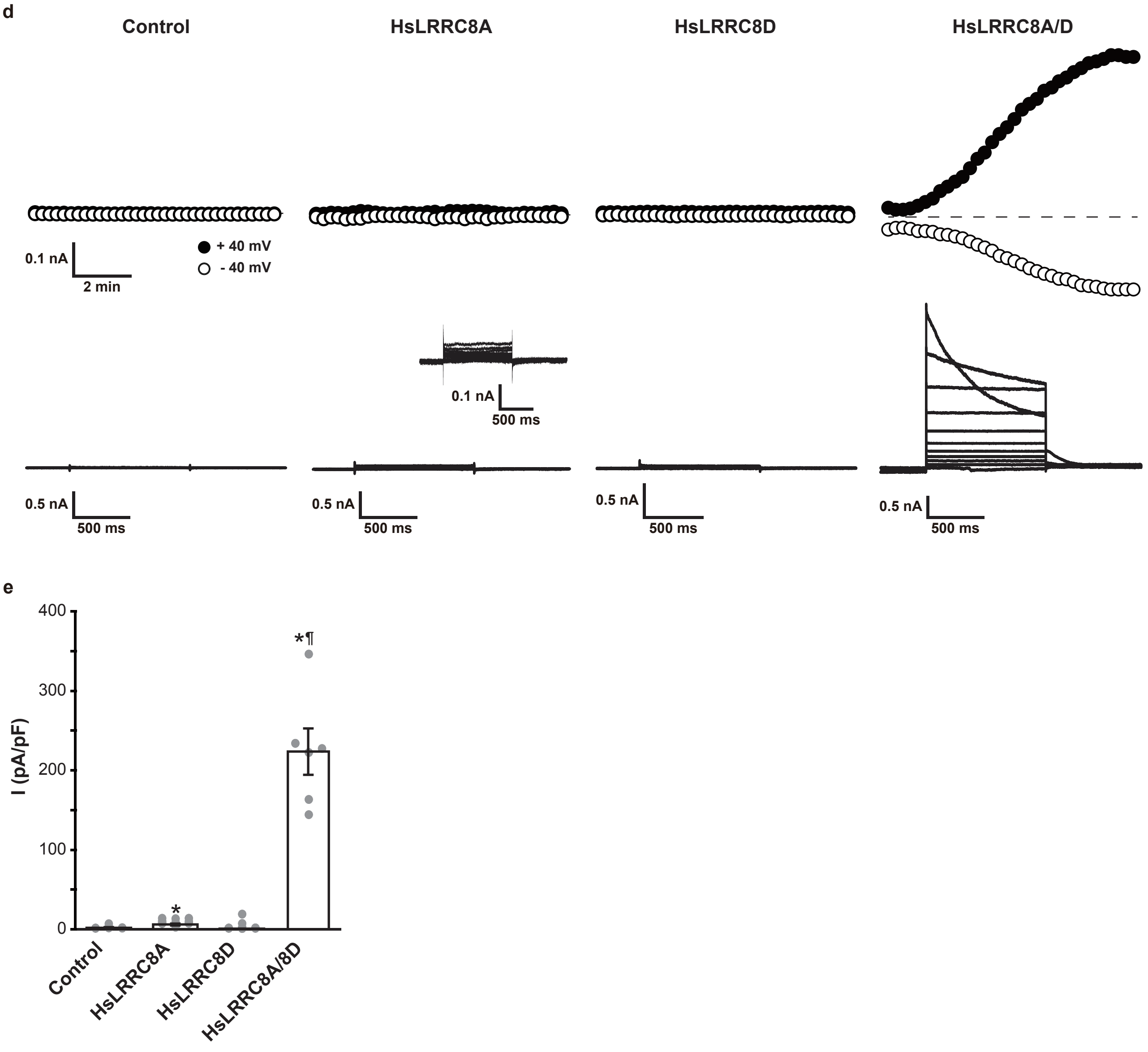

Supplementary Figure 1. Functional properties of the HsLRRC8D protein.

(**a**) Fluorescence-detection size-exclusion chromatography profiles of the HsLRRC8D (black) and HsLRRC8A (blue) proteins on a Superose 6 Increase 10/300 GL column (GE Healthcare). The void volume, EGFP-fused HsLRRC8 hexamer, and free EGFP fractions are indicated by black arrows. (**b**) SDS-PAGE of the purified HsLRRC8D protein. (**c**) List of proteins detected by mass spectrometry from the purified HsLRRC8D protein samples. HsLRRC8D (highlighted in yellow) and other detected proteins are listed in descending order of their amounts. (**d**) Representative records of swelling-induced whole-cell currents of non-transfected *LRRC8*$^{-/-}$ cells (Control), expressing HsLRRC8A wild type alone (HsLRRC8A), expressing HsLRRC8D wild type alone (HsLRRC8D), or expressing HsLRRC8A wild type and HsLRRC8D wild type (HsLRRC8A/D). Upper panels: Representative time courses of current activation induced by the introduction of the hypertonic solution into the cells. Each point represents the current recorded upon application (every 15 s) of alternating pulses of ±40 mV. Lower panels: Expanded current responses to step pulses of -100 to +100 mV in 20-mV increments at the end of the time course recordings in the upper panels. Inset: For clarity, magnified current traces of the HsLRRC8A wild type are shown. (e) Mean current densities of swelling-induced whole-cell currents recorded at +100 mV. Each column represents the mean ± SEM (n = 6-11). *, $P < 0.05$ compared to the current densities in HsLRRC8A- or HsLRRC8D-expressing cells; and ¶, $P < 0.05$ compared to the current densities in HsLRRC8A- or HsLRRC8A/D-expressing cells (one-way ANOVA analysis of variance, Tukey-Kramer test).

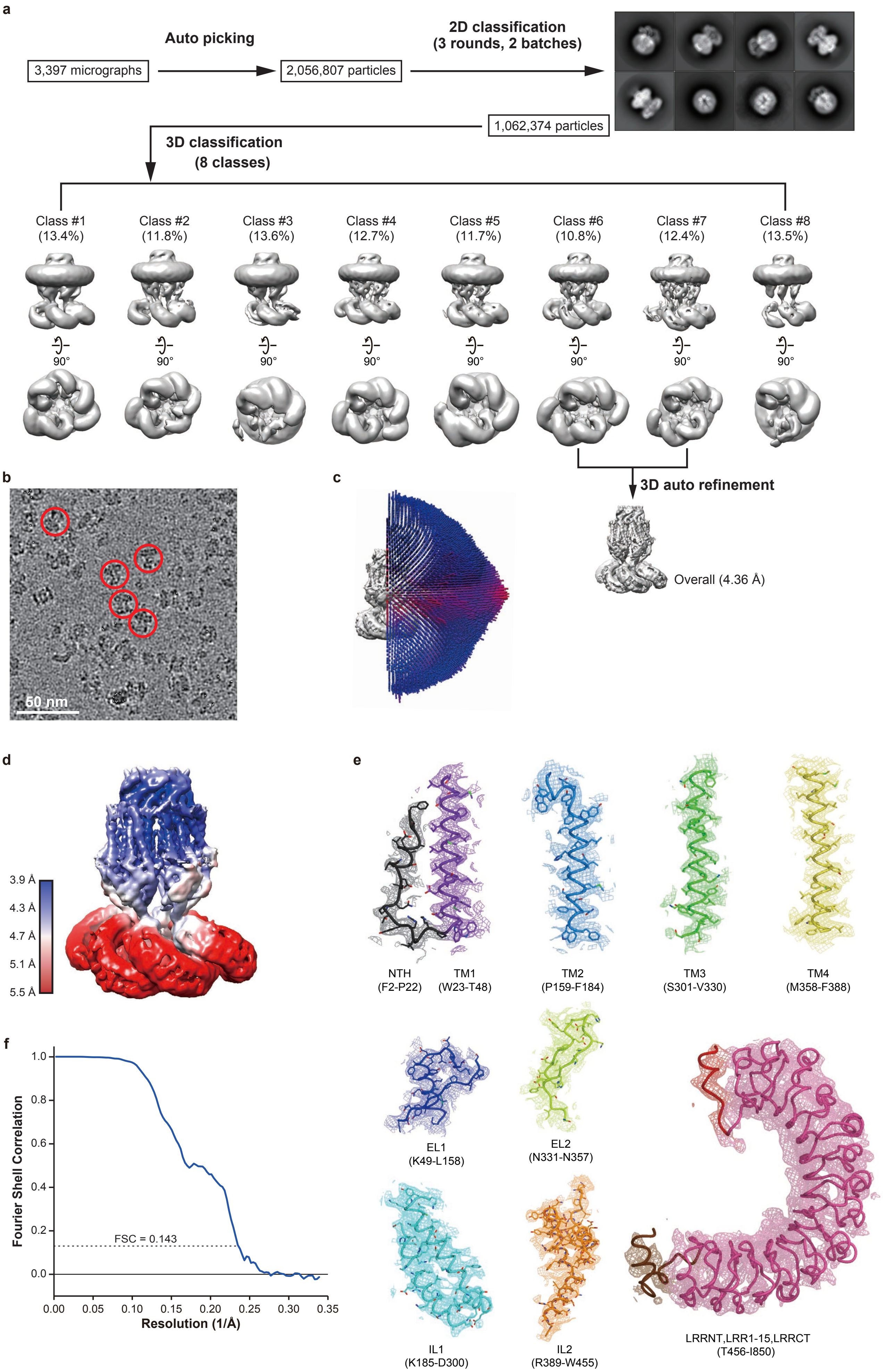
a
Auto picking
3,397 micrographs
2,056,807 particles
2D classification
(3 rounds, 2 batches)
1,062,374 particles
3D classification
(8 classes)
Class #1
(13.4%)
Class #2
(11.8%)
Class #3
(13.6%)
Class #4
(12.7%)
Class #5
(11.7%)
Class #6
(10.8%)
Class #7
(12.4%)
Class #8
(13.5%)
90°
3D auto refinement
Overall (4.36 Å)
b
50 nm
c
d
3.9 Å
4.3 Å
4.7 Å
5.1 Å
5.5 Å
e
NTH
(F2-P22)
TM1
(W23-T48)
TM2
(P159-F184)
TM3
(S301-V330)
TM4
(M358-F388)
EL1
(K49-L158)
EL2
(N331-N357)
IL1
(K185-D300)
IL2
(R389-W455)
LRRNT,LRR1-15,LRRCT
(T456-I850)
f
Fourier Shell Correlation
Resolution (1/Å)
FSC = 0.143
1.0
0.8
0.6
0.4
0.2
0.0
0.00
0.05
0.10
0.15
0.20
0.25
0.30
0.35

Supplementary Figure 2. Cyro-EM analysis of HsLRRC8D.

(**a**) Flow chart of cryo-EM data processing of the HsLRRC8D structure. (**b**) A representative cryo-EM micrograph of HsLRRC8D. Red circles indicate individual particles. The white scale bar represents 50 nm. (**c**) Angular distribution plot of particles included in the final 3D reconstruction of the HsLRRC8D structure, with C2 symmetry imposed. (**d**) Local resolution of the HsLRRC8D structure, estimated by RELION[1,2]. (**e**) Representative density maps of the extracellular, transmembrane and LRR regions. Cartoon models of the NTH, TM1-4, EL1-2, IL1-2, and LRR regions were fitted to the density maps. (**f**) Fourier Shell Correlation (FSC) curve of the final 3D reconstruction model calculated using "relion_postprocess" with masked marked 4.36 Å resolution, corresponding to the FSC = 0.143 gold standard cut-off criterion.

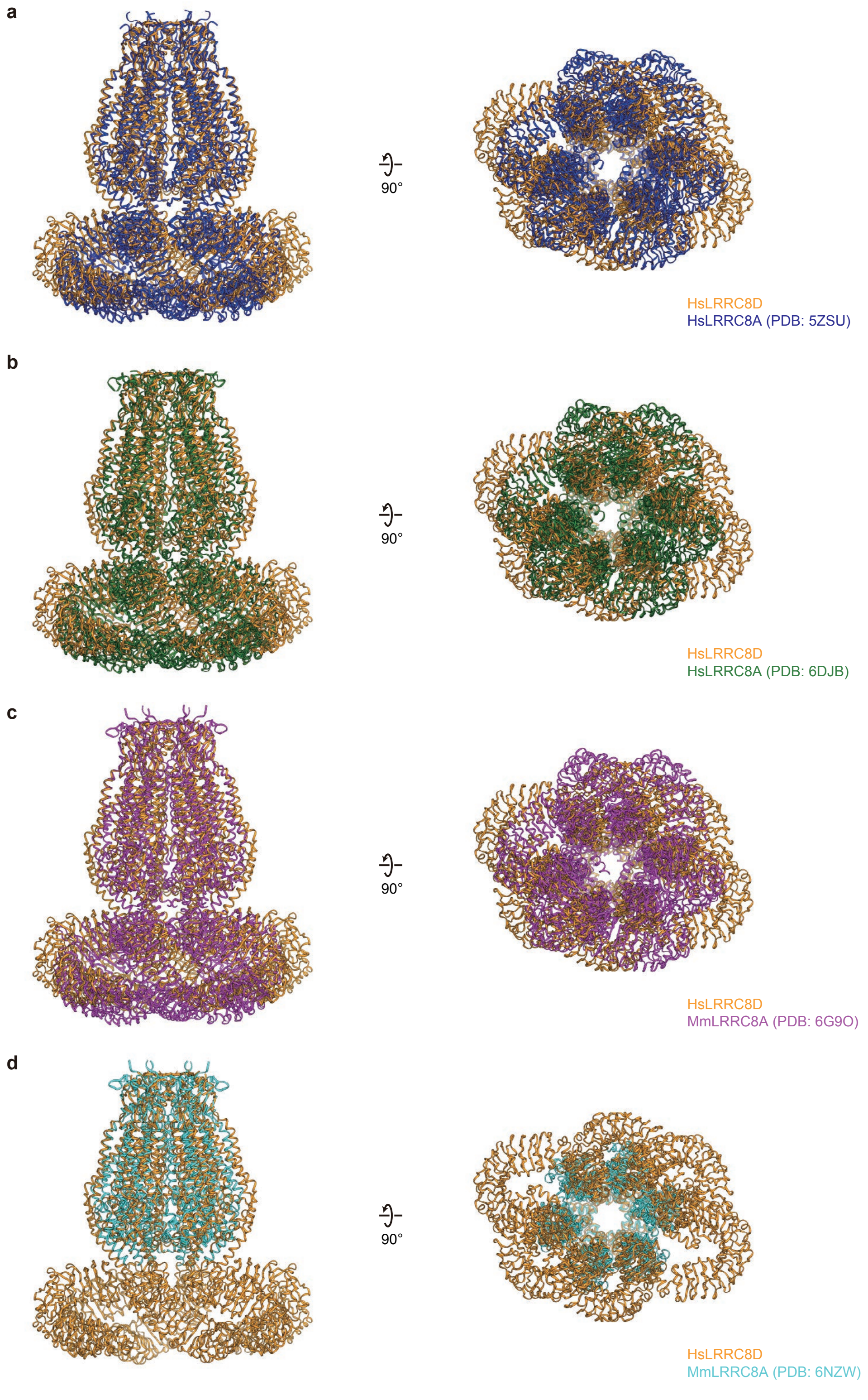

Supplementary Figure 3. Comparison of the overall structures of HsLRRC8D, HsLRRC8A, and MmLRRC8A.

(**a**) Superimposition of the HsLRRC8D (orange) and HsLRRC8A in detergent (blue, PDB ID: 5ZSU)[3] structures, viewed parallel to the membrane (left), and from the intracellular sides (right). (**b**) Superimposition of the HsLRRC8D (orange) and HsLRRC8A in detergent (green, PDB ID: 6DJB)[4] structures, viewed parallel to the membrane (left), and from the intracellular sides (right). (**c**) Superimposition of the HsLRRC8D (orange) and MmLRRC8A in detergent (purple, PDB ID: 6G9O)[5] structures, viewed parallel to the membrane (left), and from the intracellular sides (right). (**d**) Superimposition of the HsLRRC8D (orange) and MmLRRC8A in nanodiscs (purple, PDB ID: 6NZW)[6] structures, viewed parallel to the membrane (left), and from the intracellular sides (right).

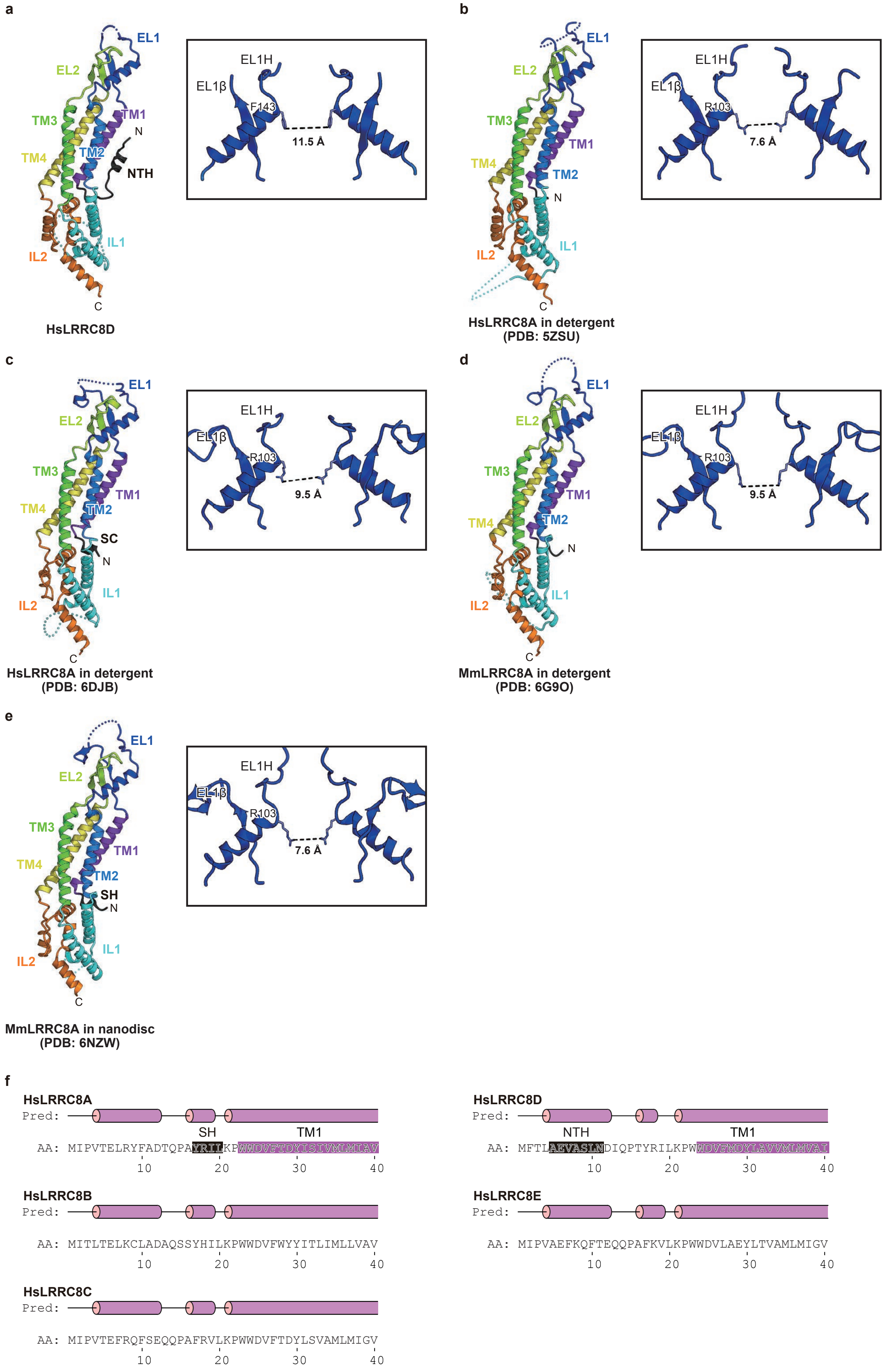
a
EL1
EL2
TM3
TM1
N
TM4
TM2
NTH
IL1
IL2
C
HsLRRC8D
EL1H
EL1β
F143
11.5 Å
b
EL1
EL2
TM3
TM1
TM4
TM2
N
IL1
IL2
C
HsLRRC8A in detergent
(PDB: 5ZSU)
EL1H
EL1β
R103
7.6 Å
c
EL1
EL2
TM3
TM1
TM4
TM2
SC
N
IL1
IL2
C
HsLRRC8A in detergent
(PDB: 6DJB)
EL1H
EL1β
R103
9.5 Å
d
EL1
EL2
TM3
TM1
TM4
TM2
N
IL1
IL2
C
MmLRRC8A in detergent
(PDB: 6G9O)
EL1H
EL1β
R103
9.5 Å
e
EL1
EL2
TM3
TM1
TM4
TM2
SH
N
IL1
IL2
C
MmLRRC8A in nanodisc
(PDB: 6NZW)
EL1H
EL1β
R103
7.6 Å
f
HsLRRC8A
Pred:
SH
TM1
AA: MIPVTELRYFADTQPAYRILKPWWDVFTDYISIVMLMIAV
10 20 30 40
HsLRRC8B
Pred:
AA: MITLTELKCLADAQSSYHILKPWWDVFWYYITLIMLLVAV
10 20 30 40
HsLRRC8C
Pred:
AA: MIPVTEFRQFSEQQPAFRVLKPWWDVFTDYLSVAMLMIGV
10 20 30 40
HsLRRC8D
Pred:
NTH
TM1
AA: MFTLAEVASLNDIQPTYRILKPWWDVFMDYLAVVMLMVAI
10 20 30 40
HsLRRC8E
Pred:
AA: MIPVAEFKQFTEQQPAFKVLKPWWDVLAEYLTVAMLMIGV
10 20 30 40

Supplementary Figure 4. Comparison of the N-terminal half subunits and pore constrictions for HsLRRC8D, HsLRRC8A, and MmLRRC8A.

(**a-e**) The N-terminal halves and pore constrictions (inset) of the HsLRRC8D subunit (**a**), the HsLRRC8A subunit in detergent (PDB ID: 5ZSU)[3] (**b**), the HsLRRC8A subunit in detergent (PDB ID: 6DJB)[4] (**c**), the MmLRRC8A subunit in detergent (PDB ID: 6G9O)[5] (**d**), and the MmLRRC8A subunit in nanodiscs (PDB ID: 6NZW)[6] (**e**). (**f**) Secondary structure prediction of the N-terminal regions of the five human LRRC8 isoforms. The prediction was performed by the program PSIPRED 4.0[7]. The actual locations of the corresponding helices are indicated by the gray boxes for NTH and SH, and the purple boxes for the TM1 helix.

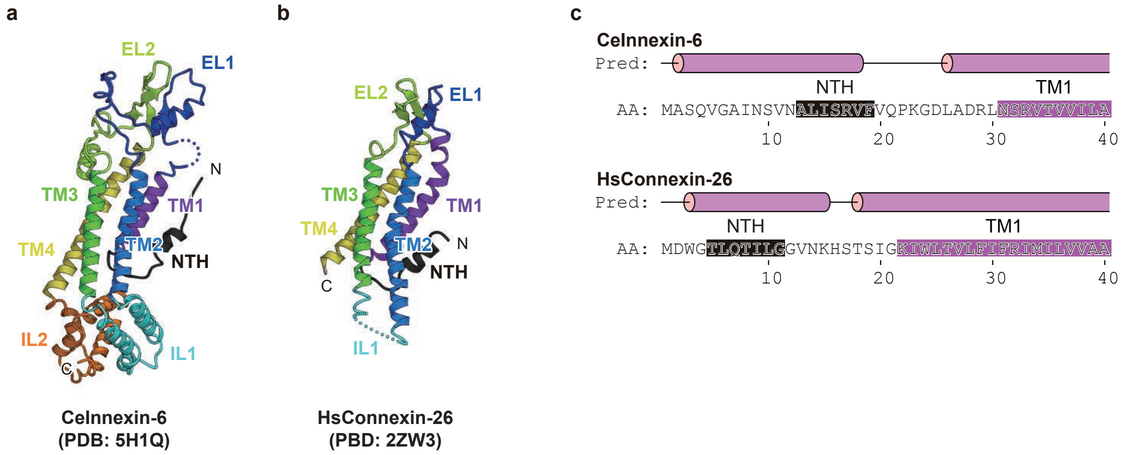

Supplementary Figure 5. Comparison of the innexin and connexin subunits.

(**a, b**) The structures of the CeInnexin-6 (PDB ID: 5H1Q)[8] and HsConnexin-26 (PDB ID: 2ZW3)[9] subunits, depicted according to the LRRC8 coloring in Fig. 1b. (**c**) The secondary structure predictions of CeInnexin-6 and HsConnexin-26, according to Supplementary Fig. 4f.

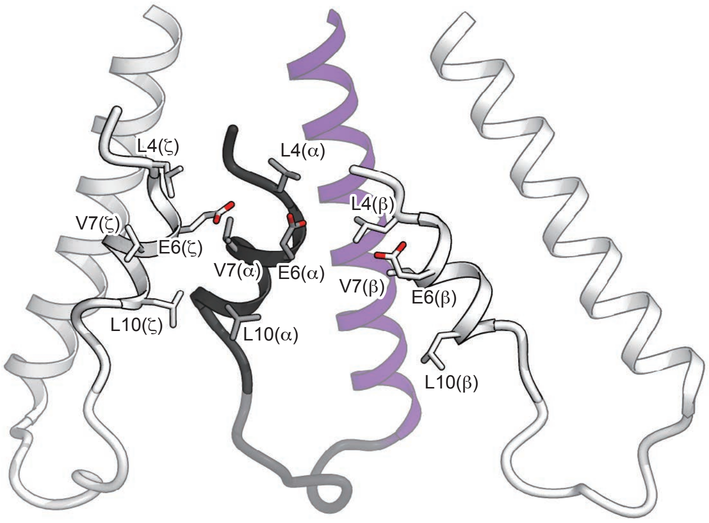

Supplementary Figure 6. Close-up view of the N-terminal region of the HsLRRC8D structure.

The N-terminal regions between the three neighboring subunits (α, β, and ζ), viewed from the central axis. The side chains of the residues involved in substrate permeability are depicted by stick models.

Supplementary Figure References

1. Scheres, S. H. W. Semi-automated selection of cryo-EM particles in RELION-1.3. *J. Struct. Biol.* **189**, 114–122 (2015).
2. Kimanius, D., Forsberg, B. O., Scheres, S. H. W. & Lindahl, E. Accelerated cryo-EM structure determination with parallelisation using GPUS in RELION-2. *Elife* **5**, 1–21 (2016).
3. Kasuya, G. *et al.* Cryo-EM structures of the human volume-regulated anion channel LRRC8. *Nat. Struct. Mol. Biol.* **25**, 797–804 (2018).
4. Kefauver, J. M. *et al.* Structure of the Human Volume Regulated Anion Channel. *Elife* **7**, e38461 (2018).
5. Deneka, D., Sawicka, M., Lam, A. K. M., Paulino, C. & Dutzler, R. Structure of a volume-regulated anion channel of the LRRC8 family. *Nature* **558**, 254–259 (2018).
6. Kern, D. M., Oh, S., Hite, R. K. & Brohawn, S. G. Cryo-EM structures of the DCPIB-inhibited volume-regulated anion channel LRRC8A in lipid nanodiscs. *Elife* **8**, 1–23 (2019).
7. Buchan, D. W. A. & Jones, D. T. The PSIPRED Protein Analysis Workbench: 20 years on. *Nucleic Acids Res.* **47**, W402–W407 (2019).
8. Oshima, A., Tani, K. & Fujiyoshi, Y. Atomic structure of the innexin-6 gap junction channel determined by cryo-EM. *Nat. Commun.* **7**, 13681 (2016).
9. Maeda, S. *et al.* Structure of the connexin 26 gap junction channel at 3.5 A resolution. *Nature* **458**, 597–602 (2009).
