# Supplementary material for: Cryo-EM structure of the volume-regulated anion channel LRRC8D isoform identifies features important for substrate permeation
Source: Commun Biol. 2020 May 15;3:240. doi: 10.1038/s42003-020-0951-z (PMC7229184; doi:10.1038/s42003-020-0951-z)
Supplement: Supplementary file 4 — Description of Additional Supplementary Files [file 42003_2020_951_MOESM4_ESM.pdf]

## Description of Additional Supplementary Files

**Supplementary Data 1.**

Source data for Supplementary Figure 1c: List of proteins detected by mass spectrometry analysis.

**Supplementary Data 2.**

Source data for Figure 4b, 4d: Patch-clamp measurements.
