# Supplementary material for: Cryo-EM structure of the volume-regulated anion channel LRRC8D isoform identifies features important for substrate permeation
Source: Commun Biol. 2020 May 15;3:240. doi: 10.1038/s42003-020-0951-z (PMC7229184; doi:10.1038/s42003-020-0951-z)
Supplement: Supplementary file 7 — file checklist [file 42003_2020_951_MOESM7_ESM.pdf]

Final submission file checklist

Should you have any questions regarding this checklist, please contact us at commsbio@nature.com
This checklist is for your own use. You do not need to resubmit this checklist.

Submission Files			
**Item**	**Permissible file format**	**Notes**	**Completed**
Cover letter	.doc, .docx, .pdf	Outline any changes to the manuscript, note whether you prefer to **opt-in** or **opt-out** of transparent peer review, and **include the title and captions for any supplementary items not included in the supplementary PDF**. This includes Supplementary Data, Supplementary Movies, Supplementary Software, etc.	☑
Editorial checklist table	.doc, .docx	A completed version of the separately attached checklist table containing editorial requests for the final version.	☑
Article File	.doc, .docx, .tex, .pdf	Only one file permitted<br>For .tex files: Please note that we cannot accept BibTeX files. References should be included within the manuscript file itself. Authors who wish to use BibTeX to prepare their references should therefore copy the reference list from the .bbl file that BibTeX generates and paste it into the main manuscript .tex file (and delete the associated \bibliography and \bibliographystyle commands).	☑
Main Figure File(s)	.pdf, .eps, .tiff, .psd, .ai, .png, .pptx	Production-quality versions of all figures, supplied as separate files. Figures divided into parts should be labelled with a lowercase bold **a**, **b**, and so on. To ensure the swift processing of your paper please provide the highest quality, vector format, versions of your images (.ai, .eps, .psd) where available. Text and labelling should be in a separate layer to enable editing during the production process. If vector files are not available then please supply the figures in whichever format they were compiled (do not save as flat .jpeg or .TIFF files). Any chemical structures or schemes contained within figures should additionally be supplied as separate ChemDraw (.cdx) files. If your artwork contains any photographic images, please ensure these are at least 300 dpi.	☑
Main Table(s)	Do not upload separately	Must be included in the Article File	☑
Supplementary Information	.txt, .gif, .html, .doc, .jpg, .swf, .mov, .xlsx, .pdf, .ppt, .wav, .csv, .zip	Can be uploaded separately or included within the Article file. Supplementary datasets should be provided as separate .xlsx files and uploaded as 'Supplementary Data' files. ** Please note that Supplementary Information cannot be changed after the paper has been accepted **	☑
Reporting summary	.pdf	A final version of the reporting summary. Be sure to include your name in the top right corner and the date. You can find a blank version of the reporting summary here: https://www.nature.com/documents/nr-reporting-summary.pdf	☑
Editorial policy checklist	.pdf	A final version of the Editorial Policy Checklist https://www.nature.com/documents/nr-editorial-policy-checklist.pdf	☑
Suggested cover image	.jpg, .pdf, .gif, .tiff, .psd	If you wish, an interesting image (but not an illustration or schematic) for consideration as a 'Featured Image' on the Communications Biology homepage. The file should be 1400x400 pixels in RGB format and should be uploaded as 'Related Manuscript File'. In addition to our home page, we may also use this image (with credit) in other journal-specific promotional material. If you submit a suggested featured image, please also include a completed image License to Publish form.	☐
